# The Duckbot: A system for automated imaging and manipulation of duckweed

**DOI:** 10.1371/journal.pone.0296717

**Published:** 2024-01-23

**Authors:** Blair Subbaraman, Orlando de Lange, Sam Ferguson, Nadya Peek

**Affiliations:** 1 Department of Human Centered Design & Engineering, University of Washington, Seattle, Washington, United States of America; 2 Biology Department, Shoreline Community College, Shoreline, Washington, United States of America; Mustansiriyah University, IRAQ

## Abstract

Laboratory automation can boost precision and reproducibility of science workflows. However, current laboratory automation systems are difficult to modify for custom applications. Automating new experiment workflows therefore requires development of one-off research platforms, a process which requires significant time, resources, and experience. In this work, we investigate systems to lower the threshold to automation for plant biologists. Our approach establishes a direct connection with a generic motion platform to support experiment development and execution from a computational notebook environment. Specifically, we investigate the use of the open-source tool-changing motion platform Jubilee controlled using Jupyter notebooks. We present the Duckbot, a machine customized for automating laboratory research workflows with duckweed, a common multicellular plant. The Duckbot comprises (1) a set of end-effectors relevant for plant biology, (2) software modules which provide flexible control of these tools, and (3) computational notebooks which make use of these tools to automate duckweed experiments. We demonstrate the Duckbot’s functionality by automating a particular laboratory research workflow, namely, duckweed growth assays. The Duckbot supports setting up sample plates with duckweed and growth media, gathering image data, and conducting relevant data analysis. We discuss the opportunities and limitations for developing custom laboratory automation with this platform and provide instructions on usage and customization.

## Introduction

By automating laboratory workflows, researchers gain speed, efficiency, and reproducibility [1]. Moreover, automated workflows help easily integrate execution of physical experiments with up- and downstream computational tools for experimental design and data analysis [2–4]. Many commercial laboratory automation systems are already available to researchers, particularly for liquid handling applications [5]. However, we argue that existing systems merely scratch the surface of what is possible with laboratory automation. Scientific workflows are often niche, and the support of all types of workflows is unlikely to be an attractive commercial proposition [1]. Therefore, to support a broad range of science workflows, we seek to develop extensible, open-source automation systems that communities of scientists can build, adapt, and extend themselves. Here we are inspired by successful open source software ecosystems [6, 7] and growing open science hardware (OSH) communities [8, 9].

Duckweeds are model plants of resurgent interest [10]. The duckweed family, the Lemnaceae, are a set of 37 species, all of which are macroscopic freshwater plants that display a reduced size as well as morphological and physiological complexity compared with all other known flowering plant species [11]. While the vegetative adults of some members of the family exhibit clearly differentiated roots and fronds, species within the *Wolffia* genus are rootless with fronds often less than 1 mm across, and with fewer than 10 discernible cell types [12]. This morphological and physiological simplicity facilitates research into many aspects of angiosperm biology, as exemplified in recent work interrogating the transcriptomics of *Wolffia australiana* [12]. In addition, small size, preference for free-floating aquatic growth, and fast growth rates relative to other angiosperms facilitate laboratory research by allowing the use of labware designed for cultivation of microscopic organisms and higher-throughput approaches. Those possibilities have been demonstrated in work characterizing physiological parameters for 39 different genotypes from across the family [13] as well as recent work developing machine automated pipelines for high-throughput studies of duckweed-microbe interactions [14]. Many species in the family flower rarely or have never been observed flowering [15]; this hampers use of traditional plant genetics approaches based on crosses. However, duckweeds have relatively small and simple genomes [12] and are amenable to existing transgenic methods [16]. In addition to these factors relevant to experimental design and methodology there are also a number of current and potential applications for duckweed species including animal feed [17], bioenergy [18] and human food [19]. In light of these advantages and applications, we are motivated to contribute to the protocols, tools, and other community resources to drive forward research about and with duckweeds.

Along with others in the duckweed research community, we identify duckweed as a promising family of organisms for use in automated experiments. Here our central research question is: how can we automate science experiments that require the manipulation of duckweed using open science hardware? Furthermore, what design goals should we include to ensure that our system is extensible by other duckweed researchers?

The Jubilee Platform is an open-source multi-motion tool-changing platform developed within our research group [20] and continually developed by a large community of users [21]. We extend the Jubilee platform with novel hardware and software to support the manipulation, feeding, and imaging of duckweed. Given our focus on automating duckweed experiments, we name the resulting configuration of machine, hardware tools, and control software “the Duckbot”. Specifically, we configured the Duckbot to support automated duckweed growth assays. Our workflow here includes a computational design tool to randomize plate layouts for a set of desired genotype and media combinations, software to manage the transfer of media and duckweed into 24 well plates, and the automated image collection of individual wells. A human operator is required to transfer the samples between the machine and growth chamber over the course of the experiment. We also provide a script to automate computational image analysis to produce growth curves.

Jubilee is designed to be extended with new tools, however its application to plant biology is a new one. In particular, there is no existing way to control the diverse array of tools like cameras, syringes, pipettes, and more that are necessary to perform an end-to-end experiment. In this work, we contribute Python modules to programatically control tools. We make use of these modules in computational notebooks to enable rapid development and iteration of experiments in code. We propose that our approach to laboratory automation–lower-level modules to control tools paired with higher-level computational notebooks to develop experiments–can help lower the threshold to automation for scientists. To investigate the promise of our approach, we ground our work here in the context of duckweed growth assays.

The power of workflow automation for duckweed phenotyping as a means to address complex multi-organismal and environmental interactions was recently demonstrated by Kose et al. [14]. They used a hardware combination of an Opentrons OT-2 liquid handling robot, bespoke transfer loops, and a robotic camera gantry within a walk-in growth chamber to carry out experiments involving 6000 duckweed units tracked over 10 days. Using a different approach, Scott et al. [22] adapted open source software to break duckweed growth assay workflows into standardized operations that can be executed with the support of just-in-time on-screen instructions.

Our focus differs from this prior work, as our emphasis is on how open science hardware can support niche automation applications and foster creative innovation, for which we consider automated duckweed growth assays as an example case study. As an alternative to modifying commercial systems, related work has contributed open source and modular systems for liquid handling [23] and other tasks [24]. In this work, we leverage the Jubilee’s tool-changing ability to demonstrate a multi-tool experimental workflow. Scientists have always built their own tools to meet research needs; we are interested in exploring how to support more custom automation workflows such as those described by Kose et al. [14].

In the results section we present details of the design of the Duckbot, requirements and guidance for local implementation, and data from example experiments. We emphasize that the results from our example experiments are not the key contribution of this work; we detail several limitations of our growth assay design below in Materials and methods. Instead, our experimental design choices showcase a range of functionality possible using our system. All code and design files can be found on our Github repositories [25, 26], with additional guides and tutorials in our documentation. As part of this work, we also developed general resources to support Duckbot builders and users including a set of tutorial videos for assembly and configuration. In the discussion section we consider the limitations and friction points of the system, and use these insights to guide opportunities for continued development. We also consider the growing ecosystem of Jubilee-compatible modules that can be combined to support laboratory research applications.

In sum, our contributions are:

- End-effectors and associated software modules for immediate use in duckweed growth assays, alongside design files and documentation [27];
- A set of computational notebooks which employ these tools to automate duckweed growth assays [26];
- A discussion of our example experimental results to motivate opportunities and limitations in extending Jubilee into other laboratory automation applications.

## Materials and methods

Here we provide information on our duckweed samples as well as specifics regarding our machine, tool, and software implementations. All code and design files can be found on our Github repository [25], with additional guides and tutorials provided on our documentation pages [27].

### Duckweed growth and housing

All duckweed lines used in our work were originally sourced from the Rutgers Duckweed Stock Cooperative [28]. We use three species of duckweed in our experiments: *Lemna minor* (clones 5500 and 8627), *Spirodela polyrhiza* (clone 7498), and *Wolffia australiana* (clone 7733). We maintained populations in 500 mL glass jars with 1.6 g/L × Schenk and Hildebrandt (SH) media [29], sourced from PhytoTech Labs, adjusted to pH 6. A small number of fronds were used to initiate new populations every 2 weeks. We maintained all three lines in a custom-built growth chamber (45x55x65 mm), under 12 hour light/dark cycled LED lighting conditions. Ramets of each line were grown to high-density in the glass jars.

We use this duckweed in a demonstrative experiment using our system to compare the growth rates of four duckweed genotypes. Our approach to duckweed cultivation weakens the validity the subsequent growth assays, as overcrowding of duckweed fronds is know to decrease growth rates [30–32]. We moreover conduct our growth assays in 24-well plates, wherein a similar problem recurs: fronds can quickly become overcrowded within wells, particularly for the larger *Spirodela polyrhiza* and *Lemna minor* duckweeds. In pursuit of high-throughput experimental systems, similar growth experiments have been conducted using *Lemna minor* duckweed in smaller 96-well plates [14]. The tradeoff between overcrowding and large-scale experimental design is therefore under active negotiation by duckweed researchers. To this end, our system can be easily modified to accommodate any other standard microplates, including larger 6-well plates or smaller 96-well plates. With this limitation in mind, we note that our central contribution with this work is not the measurement of duckweed growth rates, but rather a reconfigurable platform for scientific experimentation.

### Machine and tool implementations

We summarize the main parts of our system in Table 1, where we distinguish existing Jubilee project resources from our contributions here. Jubilee itself is licensed under a Creative Commons Attribution 4.0 Unported License (CC BY 4.0) and is certified Open Source Hardware [33]. All additional contributions we make here are released under an MIT license alongside documentation and design files, thus adhering to the Open Source Hardware Association definition of Open Source Hardware [34] The materials to build a Jubilee have been consolidated into a kit which can be purchased for general use. The Jubilee used in this work was purchased as a kit from Filastruder [35] and built ourselves. We follow existing documentation to assemble and provision the base machine [36]. We additionally created a series of videos which document the build process to benefit future researchers which can be found from our Github repository. Implementation and documentation of several relevant toolheads for plant biology already exist for Jubilee including a down-facing camera tool, 10cc syringe tool, and deck attachment to securely house six standard well-plates. We built these tools following existing documentation; for references to complete bills of materials (BOMs), please refer to Table 1.

**Table 1:** Overview of machine and tool build resources.

System Part	Existing Resources^1^	Resources Contributed in this Work^2^
Frame and Electronics	BOM, assembly instructions	Build Videos
Lab Automation Deck	BOM, assembly instructions	Flexure stage design
Pen Tool	BOM, assembly instructions	Customizable inoculation loop attachment
Syringe Tool	BOM, assembly instructions	Larger frame design for 50cc syringe
Top-Down Camera Tool	BOM, assembly instructions	N/A
Side-Facing Camera Tool	N/A	Tool Design, BOM
OT-2 Pipette Tool	N/A	Tool Design, BOM
Control Software	N/A	Usage documentation applicable to all tools

^1^ Existing resources can be found on the Jubilee Wiki [36].

^2^ New resources can be found at the Science Jubilee documentation [27]

**Fig 1:**
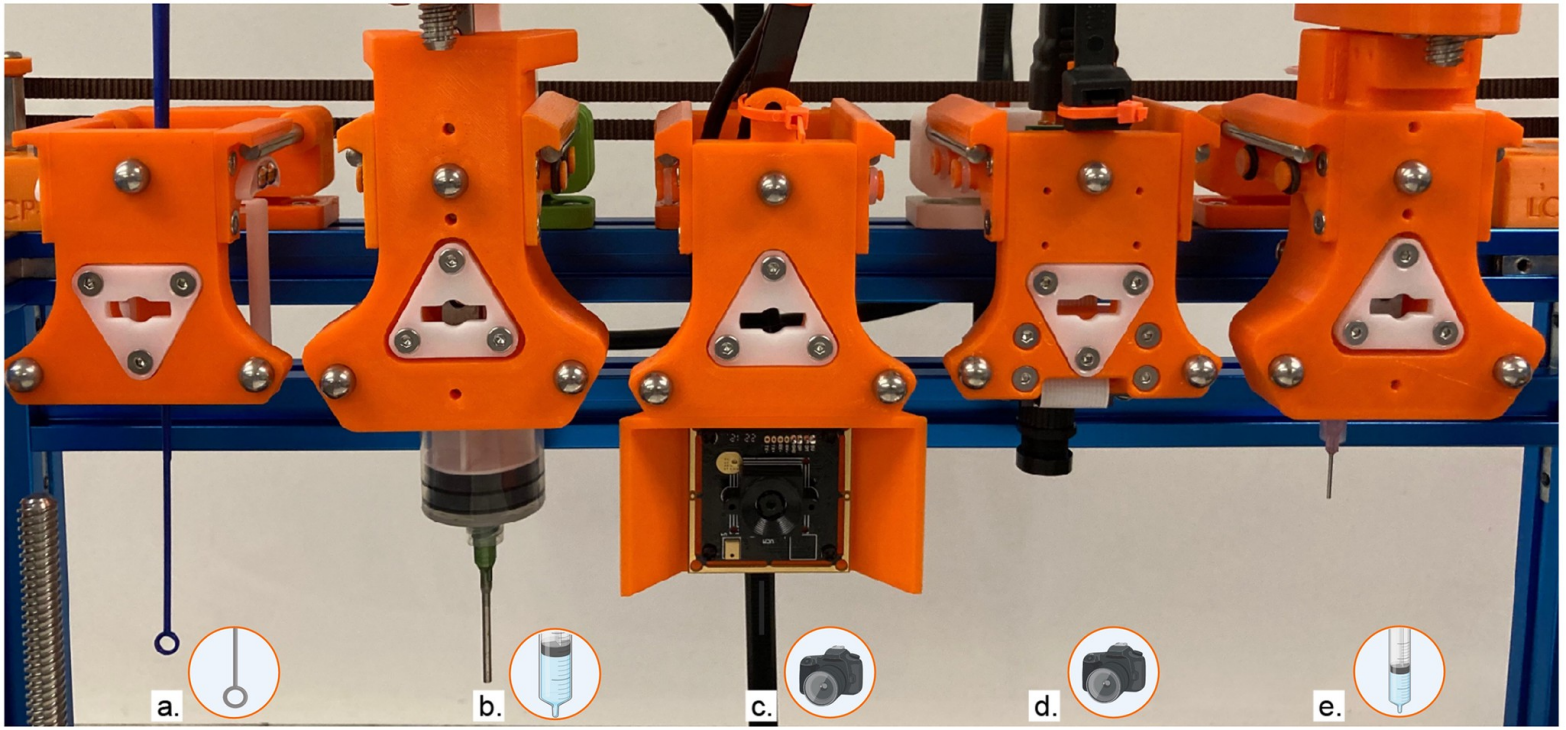
Tools designed and implemented for duckweed growth assays. (a) An inoculation loop tool. Different inoculation loops can be manually swapped into the same tool holder. (b) A 50cc syringe. (c) A side-facing camera to image parallel to the deck of the machine. (d) A top-down camera to image wells and other objects on the deck of the machine. (e) A 10cc syringe. Created with BioRender.com.

**Fig 2:**
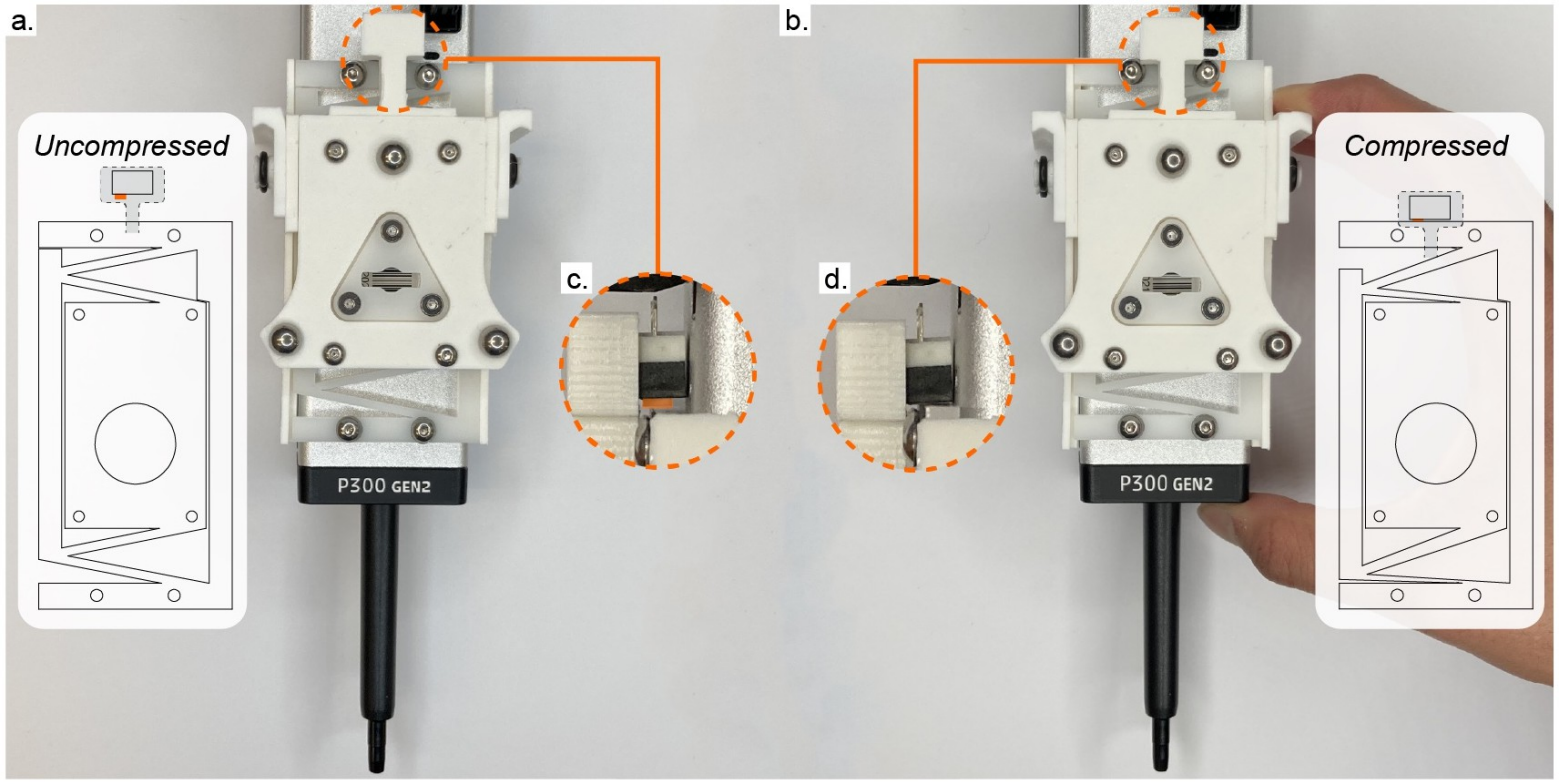
A Jubilee compatible OT-2 pipette tool. (a) The tool is designed with an external limit switch, located behind the 3D printed frame inside of the orange region of interest. (b) When pushed upwards, the toolplate is designed to translate vertically, activating the limit switch. Note the compressed flexure. (c) A magnified side-view of the uncompressed flexure. The limit switch, highlighted in orange, is not pressed. (d) A magnified side-view of the compressed flexure. The limit switch makes contact with the toolplate, signalling application of the pipette tip.

**Fig 3:**
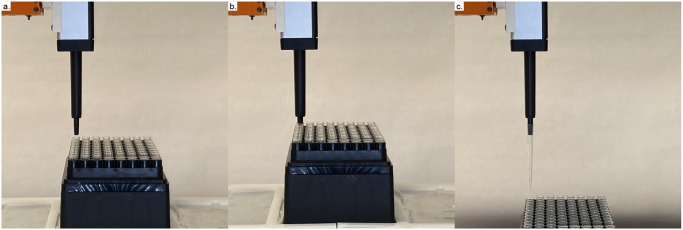
Application of a pipette tip. (a) The tool is first aligned to an available pipette tip. This process is handled by our Python modules. (b) The Jubilee platform moves upwards until a limit switch is activated. (c) The pipette tip is properly applied to the tool.

We contribute several tools relevant to plant biologists (Fig 1). For larger volume liquid transfer, we modify an existing syringe tool design. We expand the tool frame and accompanying parking post to fit the dimensions of a 50cc syringe. The tool features two attachment points to press-fit the tabs at the top of the syringe plunger and barrel. A stepper motor drives movement of the plunger. To image objects of interest which are parallel to the deck of the machine, we designed a side-facing camera tool. The frame orients a Raspberry Pi camera parallel to Jubilee’s bed plate, and features optional side panels to control lighting conditions. Finally, we present a Jubilee-compatible pipette tool which accommodates single-channel OT-2 pipettes (Fig 2). As the Jubilee platform is moved upwards into the pipette tool, the tip is press-fit onto the pipette. To know when the pipette tip has been properly applied, the toolplate is designed to translate vertically until it activates a limit switch; Fig 2c shows a magnified image of this process. The result is demonstrated in Fig 3, where the Jubilee platform is moved slowly upwards until the limit switch is activated, at which point the pipette tip is attached. Tools were designed in SolidWorks and Rhino, and fabricated using a Prusa i3 MK3 3D printer using standard PLA filament.

Beyond these tools, we also contribute modifications and attachments to existing tools. We designed a deck attachment which houses six standard sized microplates and a sharps container. The design uses laser cut flexures to account for slight heterogeneity in dimensions between manufacturers. We additionally designed a parametric inoculation loop for duckweed frond transfer. The design offers straightforward customization of inner and outer loop radius to facilitate transfer of various duckweed shapes and sizes; we provide more detail on duckweed manipulation below in Results. The loop was designed with OpenSCAD and fabricated using the same 3D printer and material. The deck attachment is made from Delrin and laser cut using a Trotec Speedy 400 laser cutter. See Table 1 for references.

### Software

Jubilee’s motion control is implemented by the Duet3 platform with extension modules to accommodate additional tools. The Duet board is connected via ribbon cable to a Raspberry Pi 4, which is used as a single board computer to run both the Raspberry Pi operating system as well as the Duet Software Framework. This permits machine control via the Raspberry Pi and exposes USB and serial interfaces to incorporate sensors and cameras. The Duet offers a graphical web interface to send G-code, the most widely used machine control language, to the machine. By default, G-code commands can be sent via file uploads, virtual buttons, or individual commands. We extend this functionality by developing software libraries for controlling the Jubilee using serial communication over a wired USB connection. Our software is written in Python and compatible with Python 3. Python is a well supported language for biology applications and is commonly used for data analysis and management. Serial communication enables the same code to be easily run from any connected computer; this offers advantages for computationally intensive applications in which the Raspberry Pi becomes a limiting factor.

Our software contributions are divided into two code repositories. First, to control the Jubilee motion platform and its tools programatically, we contribute an application-agnostic Python package called science_jubilee [25]. In addition to a main machine control module which facilitates movement of the motion platform, each tool type is accompanied by a corresponding Python module. For example, a syringe module exposes methods for aspirating and dispensing media; a lab automation deck module is used to navigate around the wells of microplates installed on the machine; a set of camera utilities provides a wrapper around existing Raspberry Pi camera software to easily integrate image processing and collection alongside machine control. The code moreover supports the use of custom labware and uses the same labware definitions as OpenTrons for cross-compatability. These libraries lower the barrier to developing custom automation routines. While the software has been developed in alignment with our case study to automate duckweed growth assays, these libraries provide a set of primitive actions generalizable to other use cases. Users can at any time write custom code which extends existing functionality for a new use case. Alongside the code, we contribute tutorials and guides for using science_jubilee at our documentation pages [27]. This documentation includes background information and resources to build the Jubilee motion platform, instructions to assemble the tools discussed in this work, guides for using the codebase, a complete code reference, and steps necessary for others to develop and share their own tools and control software for use by others. science_jubilee is therefore the main resource to be used by others interested in implementing a laboratory automation system using Jubilee.

To scaffold semi-automated duckweed growth assays, we make use of these software libraries in a set of Jupyter computational notebooks. A flexible programming interface with low barriers to entry is crucial to promote broad engagement for a laboratory automation platform [37]. Computational notebooks permit traditional code to be written and distributed alongside plain language explanations. As a result, they have achieved widespread adoption for biology applications [38, 39]. Jupyter notebooks are particularly appealing for biology automation contexts, as we might expect professional biologists to be comfortable writing Python code but have limited prior experience working with computer-controlled machines like Jubilee. We organize our growth assay automation routines over a set of Jupyter notebooks which provide scoped code blocks side-by-side with on-boarding information in natural language. In addition to the core set of growth assay notebooks, we also provide auxiliary notebooks to help guide the user through various machine and tool calibration steps. These notebooks can be found in our duckbot code repository [26], which in turn relies on the science_jubilee code described above. The notebooks therefore serve as an example use case of science_jubilee. In lieu of extensive external documentation, we use the Jupyter notebook environment to interleave prose and code so that these notebooks can serve as a reference for others.

## Results

**Fig 4:**
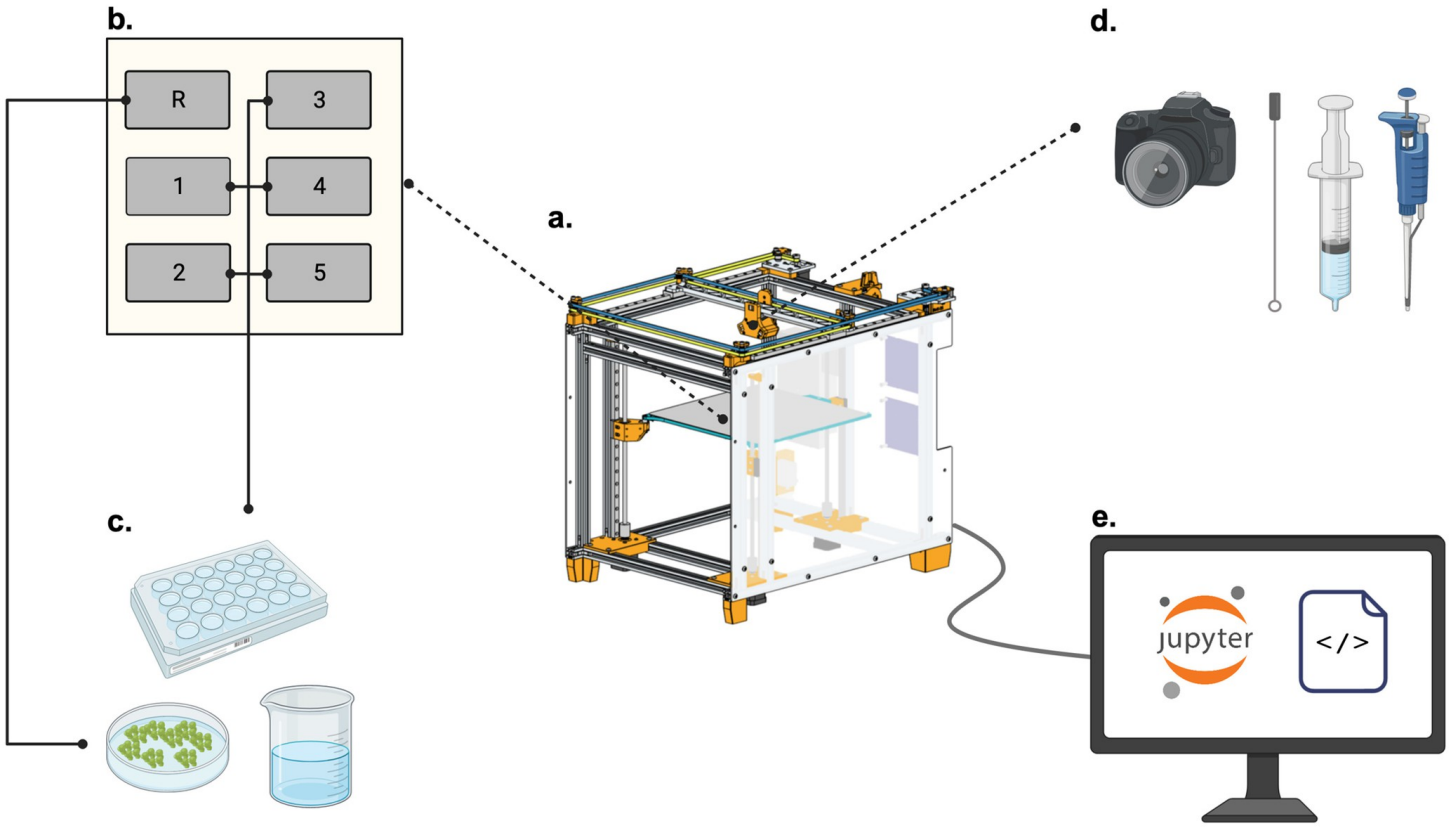
Graphical overview of the automated growth assay workflow implemented by the Duckbot. (a) The Duckbot is a Jubilee multi-tool changing motion platform outfitted with tools and associated software for duckweed handling. (b) A lab automation deck attachment is installed on the machine which supports six standard size well plates to be inserted. By default, our software assumes the first available position will be used for media reservoirs, marked R, and the other positions are available for well plates. This setup is configurable. (c) Relevant media reservoirs for our duckweed assays are petri dishes filled with duckweed or growth media. We use 24-well plates as our primary labware in our examples. (d) The Duckbot is outfitted with tools including a top-down camera which images the bed plate, a side-facing camera, an inoculation loop holder, a 10cc syringe, a 50cc syringe, and an OT-2 P300 pipette. The Duckbot can switch between these tools; inactive tools are parked along the back rail of the machine. (e) We scaffold our growth assay with a set of Jupyter notebooks which make use of our custom machine and tool control libraries. The notebooks are run using a Raspberry Pi. Created with BioRender.com.

**Fig 5:**
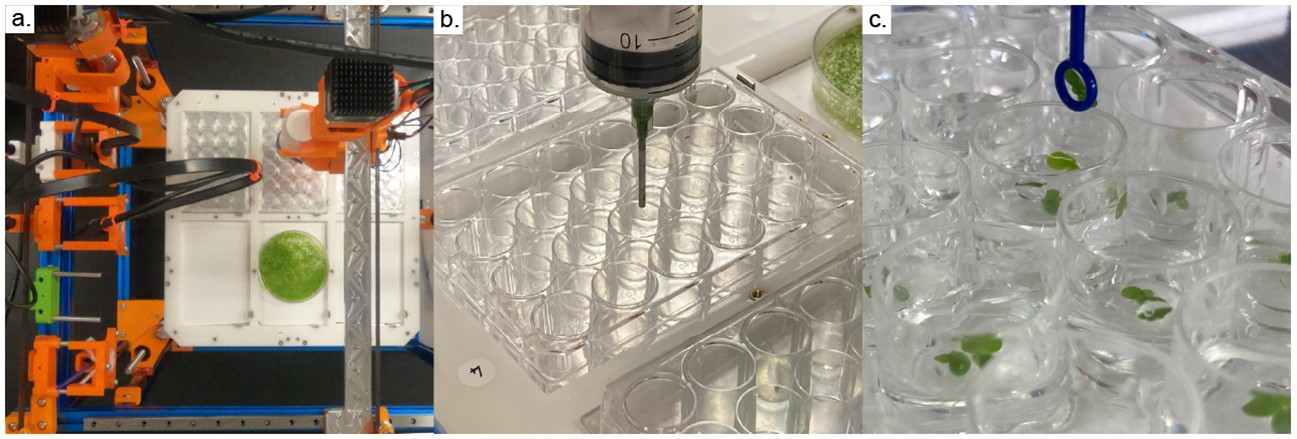
Overview of the growth assay workflow. (a) An overhead image of the Duckbot. Tools are parked along the rear rail of the machine. A deck attachment houses well plates and media reservoirs; tools can be picked up and changed to manipulate materials installed in the bed. (b) A 50cc syringe tool fills wells with relevant media given an experimental definition. (c) Duckweed fronds are picked up and transferred using inoculation loops, among other tools.

Our demonstrative case study automates duckweed growth assays using the Duckbot (Fig 4). We structured the growth assay workflow across four Jupyter notebooks which guide the operator through (1) defining the experimental parameters, (2) setting up plates including transfer of media and duckweed, (3) imaging plates, and (4) data analysis (Fig 5). These steps assume that the machine and requisite tools have been assembled and calibrated. All code is open-source and can be found at the science_jubilee and duckbot repositories discussed earlier. We step through the notebooks in this section, providing design justifications and results from our case study. We highlight that the notebooks presented provide an example workflow; we consider other applications in the discussion.

As shown in Fig 4, the Duckbot can be thought of as a workflow design space in which hardware (tools, decks), software (Jupyter Notebooks, tool modules), and wetware (duckweed, media) can be combined in various combinations to achieve user goals. A custom deck attachment divides the standard deck into 6 identical rectangular slots, each the size of a standard laboratory microplate (Fig 4b). The deck attachment makes use of a flexure-based design to account for slight labware heterogeneity between manufacturers. To standardize positions, we define the first available position as index zero, to be used to place source material such as media or duckweed. The remaining 5 positions hold labware to receive material. We use 24-well plates for our duckweed growth assay. This could be modified by the operator for other applications, and other well plates (e.g. 12- or 96-well plates) can also be used. We include calibration routines in the form of Jupyter notebooks for new labware in our code repository.

The first computational notebook is a simple computational design tool to randomize the plate layouts for a set of desired duckweed genotype and media combinations. Users are prompted to enter experimental parameters. This includes a name to uniquely identify this experiment, a list of the duckweed genotypes to be used, a list of the media to be used, and a number of replicates for each condition. Executing the subsequent code blocks will randomize the location of each replicate around the minimum number of well plates required. The resulting experimental configuration is saved in a human-readable JSON file format which allows the user to manually inspect and edit the experiment if needed. All data is saved in an organized file structure uniquely identified by the experiment name. This allows straightforward data backup via version control software (e.g. git, Github), or other cloud synchronization software (e.g. Google Drive).

**Fig 6:**
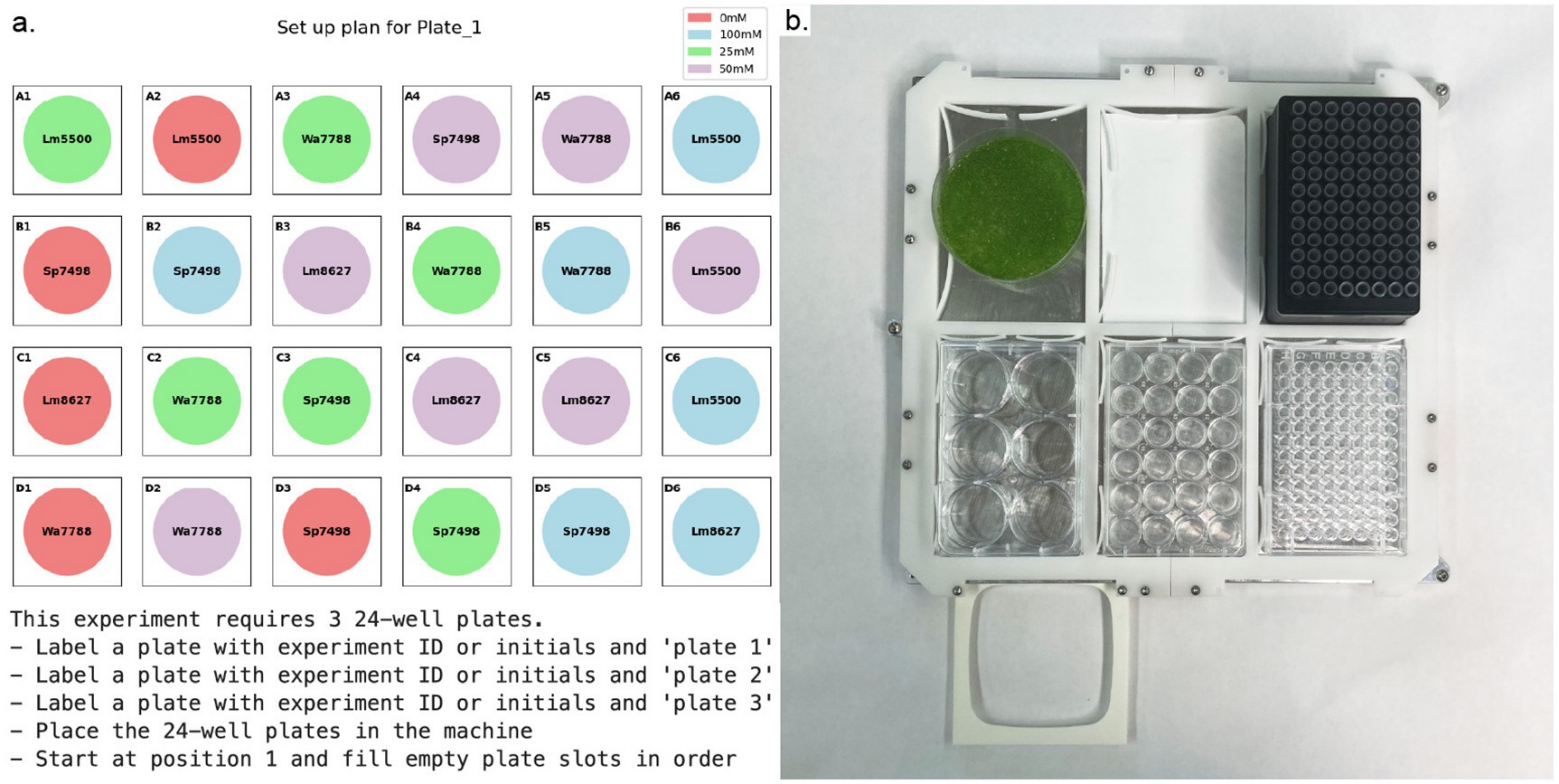
Setting up experimental well plates with the Duckbot. (a) The software visualizes the experimental setup. The first (of three) 24-well plates is shown, with different media and duckweed strains labeled. Given the experimental setup, the Jupyter notebook will guide the operator through machine setup. In this case, the three well plates are loaded into the relevant bed plate position. (b) The lab automation deck offers six slots which can be installed with various labware. Shown here is a reservoir of duckweed, an OpenTrons pipette tip rack, and 6-, 24-, and 96-well plates. A 3D printed attachment houses a sharps container.

The second Jupyter notebook scaffolds plate setup. Setup includes both media transfer and duckweed transfer. Media transfer is achieved with a 50 mL syringe controlled by a stepper motor. For the syringe tool, we extended the 3D printed frame of an existing open-source syringe tool design to allow for the insertion of a larger syringe (see Table 1). The needle or the entire syringe can be swapped out manually by the user in between media transfers if desired. For lower-volume, more precise liquid handling needs, the pipette tool can be used. Using the experimental configuration file, the Jupyter notebook will prompt the user to load the relevant number of well plates into the machine (Fig 6a). Then, the notebook will prompt the user to insert a reservoir of the first media into the reservoir position of the bed plate. After manual confirmation, the machine will automatically aspirate and dispense a given volume of media into each relevant well. The volume is configurable in software. Upon completion of the first media type, the software will prompt the operator to swap in the second media solution. The bed plate is automatically lowered to accommodate physical configurations on the machine.

**Fig 7:**
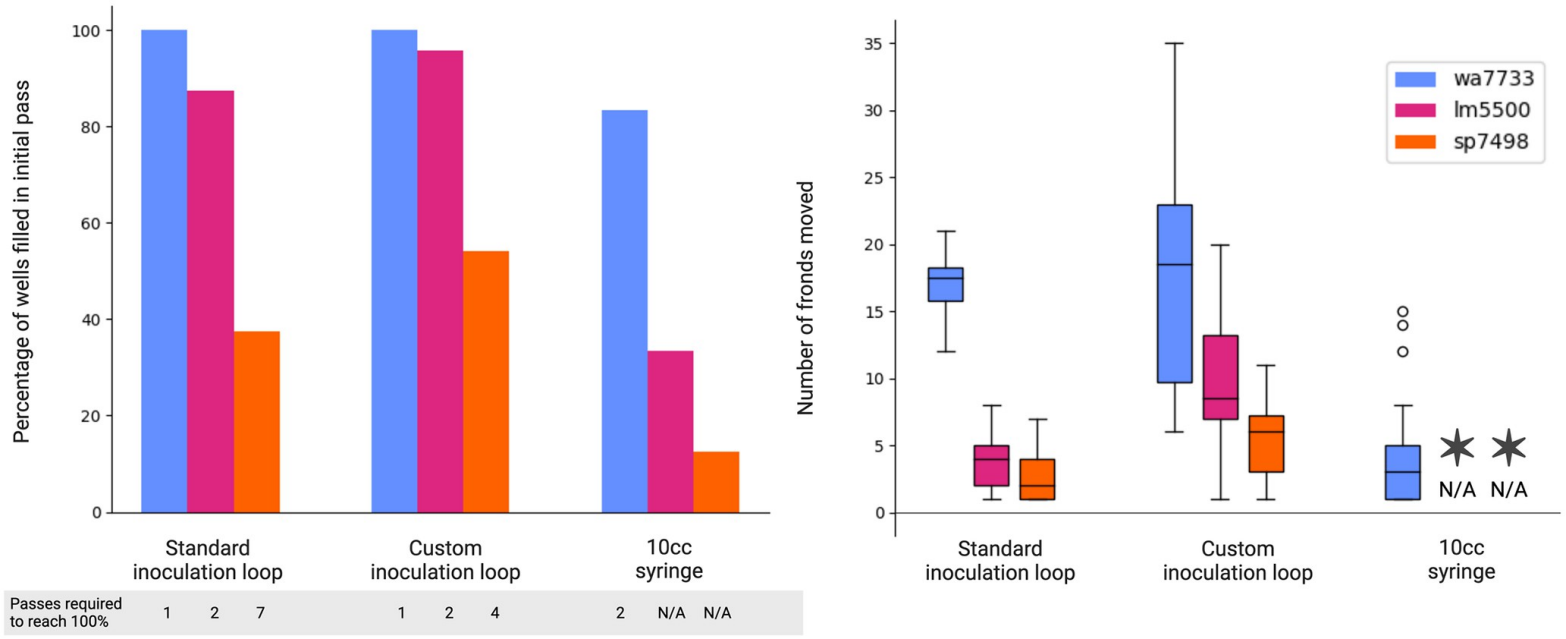
Evaluating duckweed transfer options. Three tools were tested including a standard inoculation loop, a custom 3D printed inoculation loop whose loop is at a right angle to the handle, and a 10cc syringe. Inoculation loops were simply dipped into the duckweed reservoir; the syringe was used in conjunction with the camera tool to identify an individual frond to aspirate. Each tool can be used as a part of a closed loop wherein transfer success is confirmed using the camera tool, and automatically retried on failures. Created with BioRender.com.

We evaluated a number of options to achieve duckweed frond transfer, illustrated in Fig 7. Our initial approach was to rely on a 10 mL syringe with a 20-gauge needle, programmed to move to the position of fronds in the source plate, aspirate to vacuum-attach the frond to the syringe aperture, and then to move to the destination well and dispense. This approach proved very sensitive to the exact height (z-position) of the water column in the source container, as well as exact positioning of the syringe over the center of the frond. In addition, success rates differed greatly among the three species we trialed, with almost no successful syringe transfer for *Spirodela polyrhiza*. This is likely due to the heavy, multi-frond ramets and entangled root systems.

Syringe transfer success rates can be improved with addition of a code block to iterate through multiple rounds of transfer. The camera tool collects an image of each destination plate after the first transfer round to identify wells that are still empty, and a second attempt is initiated for those wells. This can be repeated indefinitely, but with a corresponding increase in the duration of the transfer process. An inoculation loop can also be used for transfer. The inoculation loop can be dipped into the source plate and then transferred to the destination well. We found improved success rates compared to syringe transfer, but in most cases multiple ramets were transferred into each destination well. Multiple ramets might be beneficial or undesirable for different applications. Using the inoculation loop, we still observed low rates of successful frond transfer for *Spirodela polyrhiza*. To compensate for their heavier structure, we also 3D printed custom inoculation loops of various size and joint angles. A 90 degree loop proved the most successful in transferring *S. polyrhiza* ramets. In addition to the automated options, we also make available just-in-time text instructions to guide a human user to manually transfer fronds using tweezers from source to destination. Following the results presented in Fig 7, we use a standard inoculation loop for the tranfer of *Lemna Minor* and *Wolffia australiana* and a custom incoluation loop for *Spirodela polyrhiza* in the growth assay presented here.

**Fig 8:**
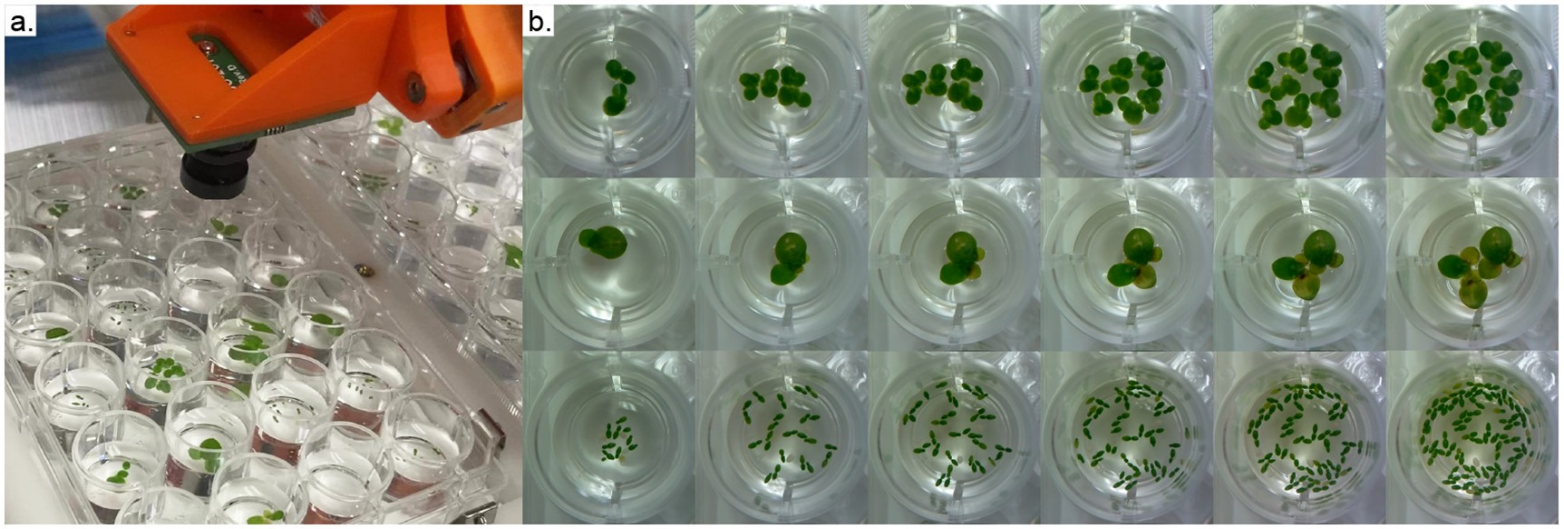
Image collection and analysis. (a) The top-down camera tool is used to image individual wells. (b) Example images over a 1-week period track the growth of *Lemna minor* (top), *Spirodela polyrhiza* (middle), and *Wolffia australiana* (bottom).

The third Jupyter notebook scaffolds image data collection. Unlike the first two notebooks which will usually be run once per experiment, data collection will be run multiple times. The Duckbot supports the collection of a top-down image time-series to calculate growth rates derived from frond surface area. We used a simple frame to hold a Raspberry Pi camera perpendicular to the bedplate, with the camera connected to the Raspberry Pi via an HDMI-to-CSI cable (Fig 8a). During data collection, the user launches a Jupyter notebook that prompts them to insert all experimental well plates in the machine. The camera frame accommodates both a manual focus camera with interchangeable lenses, as well as a camera with programmatically adjustable focus (for model numbers and links, see the materials in Table 1). Images are taken of individual wells. It takes approximately a minute to individually image each well of a 24-well plate. For higher-throughput imaging, the user can also drop the bed plate down and image all well plates at once, which can be parsed into individual wells after the fact. We opt for individual well images in our distribution to promote the highest quality data.

Images are saved using the date, experiment name, plate, and well as the filename. This naming convention allows images to be easily retrieved and categorized. User modifications to image analysis processes are particularly straightforward since they do not require any hardware modifications. To speed up the analysis of the hundreds of images collected in an experiment, our default image processing pipeline is to first convert the RGB image to a HSV (hue-saturation-value) channel image. We then apply a binary threshold based on the saturation channel; the lighting conditions when operating the machine are consistent, but this value will need to be adjusted up-front for use in other settings. We then identify the contours of this image, filtering out contours with very small areas or irregular shapes (i.e., non-round, quantified by the ratio of the contour perimeter to area) to ensure we capture only the duckweed fronds and not lighting artifacts. The resulting imaging pipeline avoids tedious hand-tracing and tuning of green pixel values which may differ depending on duckweed species, health, and lighting conditions.

As a case study, we used the Duckbot to design and execute an experiment to compare the growth rates of four duckweed genotypes (*Spirodela polyrhiza* clone 7498, *Lemna minor* clones 5500 and 8627, and *Wolffia australiana* clone 7733). We investigate growth rates under four media regimes (0, 25, 50 and 100 mM NaCl supplements into 0.5x Schenk and Hildebrandt growth media) with four replicates of each combination. The resulting experiment necessitated 64 wells, and was conducted using three 24-well plates over 10 days. The Jupyter notebooks described above were followed with no modifications. The most time-intensive operator engagement with the machine was required on day 0, during plate setup. Subsequent days required only loading plates into the machine for autonomous image collection.

**Fig 9:**
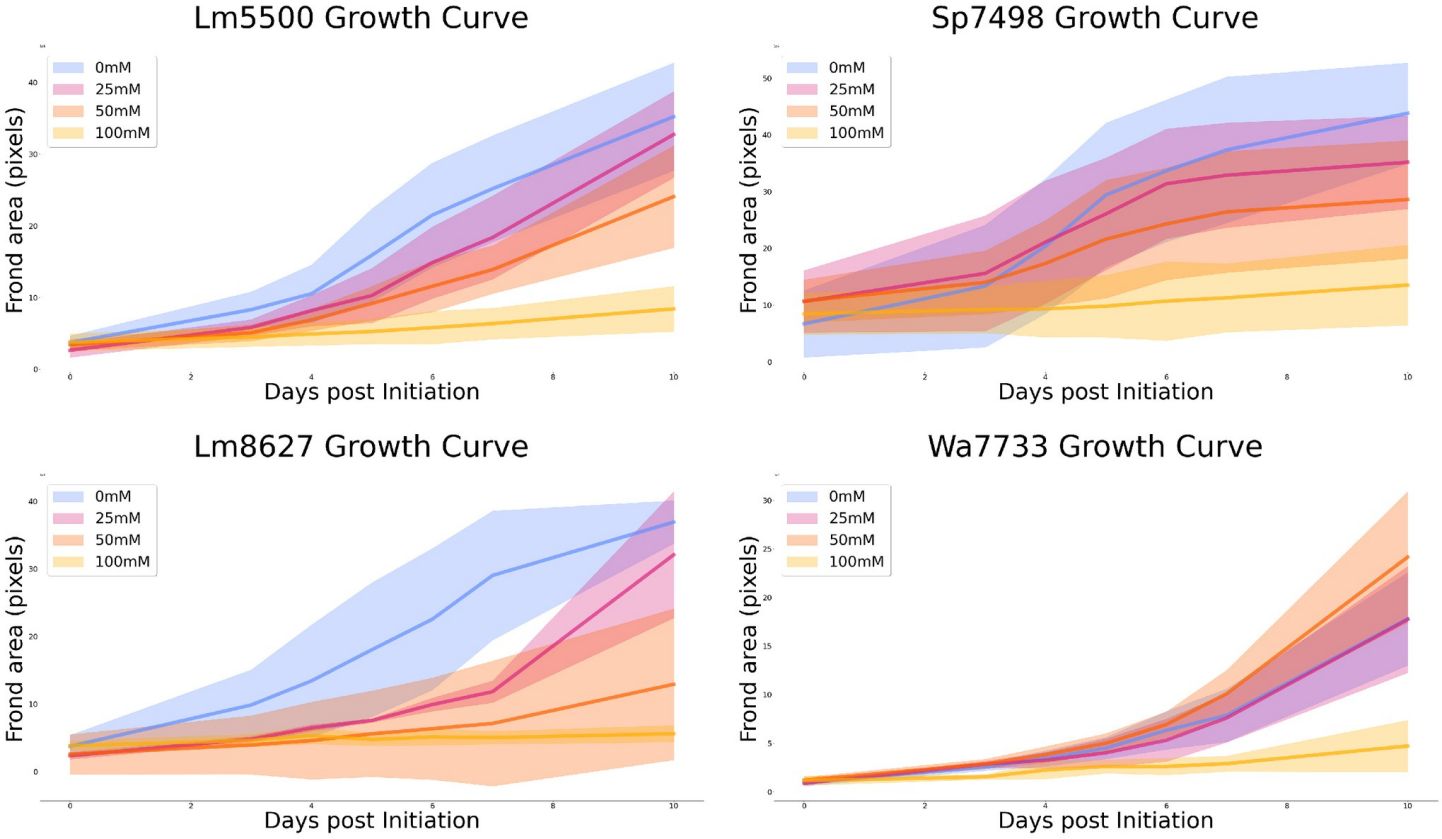
Growth curves for each duckweed genotype. Frond area over time is presented for each of our four duckweed genotypes under each media regime.

Fig 9 presents growth curves generated by our final Jupyter notebook. A total of 448 images were collected. From this data, frond area in pixels is plotted over the ten day growth assay. In alignment with prior work on duckweed salt tolerance [40], we observe that growth rates decrease with an increase in salinity with all genotypes exhibiting the least growth under the 100 mM NaCl condition. *Wolffia australiana* experienced similar growth under 0, 25, and 50 mM conditions, however we note that no significance tests were performed.

We reiterate that the data presented in Fig 9 is not the central contribution of this work. Overcrowding of fronds in the wells over the course of the growth assay, as well as during cultivation (as discussed in Materials and methods), limit the validity of the results here. Rather, we present the results of our demonstrative experiment to illustrate our system’s functionality. In the following discussion, we elaborate on future possibilities enabled by an automation platform which integrates experimental design, machine execution, and analysis.

## Discussion

Overall, we contribute (1) the Duckbot, a set of hardware and software tools for liquid handling, imaging, and frond transfer applications with thorough documentation, and (2) insights into the automation of niche laboratory workflows.

Our work adapting an open source tool-changing platform to support duckweed growth assays contributes tools relevant to other scientists. In particular, we contribute syringe, pipette, camera, and inoculation loop tools for liquid handling, imaging, and frond transfer applications. Our tool designs and software are open-source and distributed with the instructions necessary to build them (see Table 1). These tools enabled semi-automated experiments from experimental design through data analysis. Our experiences using the system suggest that Jupyter notebooks complement many facets of existing biology workflows. We retain easy access to commonly used Python libraries and promote integrated data analysis. This is particularly well demonstrated through the automated imaging and subsequent analysis of duckweed growth. The Duckbot provides imaging and motion capabilities necessary for automated data collection. The lack of easy methods for high-throughput duckweed imaging is a current barrier in experimental design [41]; the Duckbot provides infrastructure to support such tasks. Moreover, the computational notebook environment supports inline documentation for operators to familiarize themselves with the system. While computational notebooks are commonplace in the software workflows of scientists, their use for machine control is less common. Future use of the machine by professional biologists with limited prior automation experience can help elaborate the use of computational notebooks for machine control.

Our case study into niche laboratory automation moreover suggests future possibilities for novel experimental design. Refining each step of our growth assay could enable efficient, high-throughput experiments with the Duckbot. Of interest to us, however, is how the system can be reconfigured and customized to enable new experiments. For example, the straightforward computer vision techniques demonstrated in our data analysis can be integrated into experiments to differentially manipulate samples based on the overall frond area or rate of growth. Moreover, scientists might create feedback loops such that the results of one experiment drive the design of a subsequent one. The system can thus be customized to efficiently sweep several parameters.

The extensibility of the system means that it can be adapted for entirely different experiments. Beyond software customization for an individual application area, new tools can be added to accommodate new workflows. In the case study presented, for example, we were able to accommodate larger volumes of liquid transfer by adding a 50cc syringe tool. We highlight this as a main distinguishing technical factor from existing systems. Rather than design a one-off system, the Duckbot prioritizes hardware customization. The duckweed growth assay presented in this paper offers one example of this process; we see opportunity for a foundational set of laboratory tools to be developed and shared through future use with the system by professional biologists. We reflect on future application areas below.

In contributing tools for custom lab automation, we acknowledge several limitations of our case study. First, our system does not ensure a sterile experimental environment. The machine design might be adjusted to remove the side electronics panel such that the machine can be operated inside of a laminar flow hood. Our duckweed growth assay used only water. More dangerous materials would require additional safety considerations. Higher-throughput experiments which require the machine to run continuously without the presence of a human operator would require additional error detection and failsafes to ensure the machine does not damage itself or valuable chemical samples.

More broadly, our case study surfaces trade-offs and opportunities in building custom hardware for laboratory automation. In particular, we identify a tension between modular possibilities and technical expertise. Building the system is an investment. In almost all cases, onboarding for a commercial machine will be faster, allowing researchers to devote time to experimental design and data collection rather than building and debugging a machine. Cost-savings may be adequately large to offset the loss of time and attention, at least for some systems [42]. The most promising benefits, however, are for highly customized applications with no existing commercial solution. Of course, customization requires existing skill in computer aided design software to design new tools, hardware and electronics knowledge to modify and setup a tool, and programming skills to control the tool. However, we also note that the Duckbot and its constitutive parts are surrounded by large online communities. Python has a large user base of scientists, with Jupyter notebooks increasingly used as a tool to share and document open science [43]. Open electronics have seen less uptake in biological research and in science more generally [44], and there are therefore fewer domain-specific resources available catered for biologists. Oellermann et al. argue that this is in part due to the fragmented nature of existing open-electronics user communities which hinders knowledge exchange. The Duckbot benefits from existing open-source communities around the Raspberry Pi, Duet electronics boards, and the Jubilee motion platform. It thus makes for a promising site for building and exchanging the knowledge necessary for custom lab automation.

**Fig 10:**
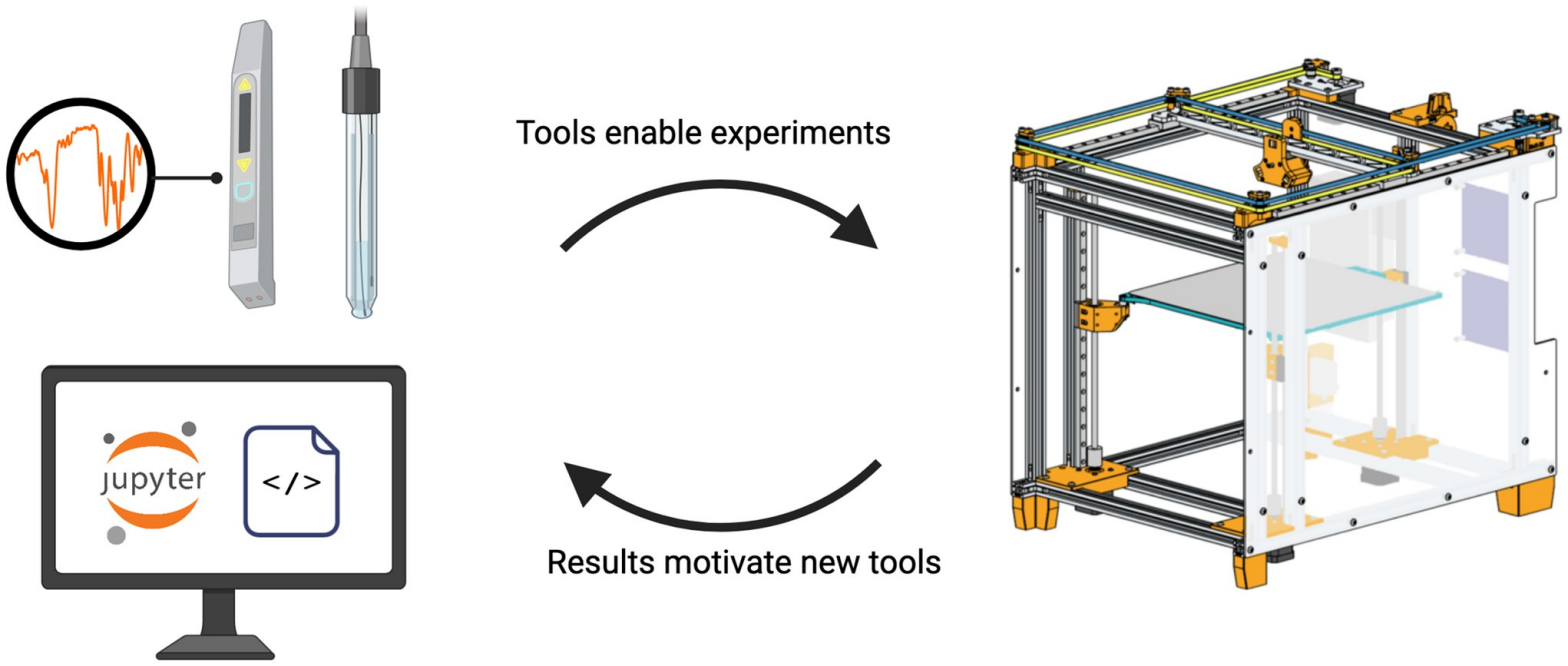
Jubilee as an open platform for automation of laboratory research. New tools and a software can be integrated into the tool-changing system to enable novel experiments. The experimental results can then be used to motivate new tool development for subsequent experiments. The resulting tools and software can be shared back into the community for reproduction and extension by others. Created with BioRender.com.

In cultivating a community of scientists using the Duckbot for laboratory automation, we envision the diversity of laboratory-relevant tools to grow alongside the user base (Fig 10). New tools can support a host of different experiments. For example, a variety of probes can be immersed in media using the same Jubilee tool as our inoculation loop; a sonicator tool has been elsewhere integrated into high-throughput workflows using Jubilee [45]. Augmented deck attachments can provide heating, cooling, and stirring capabilities. Such elements can be assigned in different combinations among the six available slots. The ability to switch between tools, combined with a programmatic approach to defining experiments, is consonant with visions of self-driving laboratories [46]. Moreover, experimental results motivate the development of new tools. The resulting tools and associated control software can be shared with the community for use by other scientists.

We identify several future lines of research using our system both for duckweed research and beyond. Our case study focused on a demonstrative, lower-throughput example. Higher throughput experiments can be run by utilizing 96-well plates in conjunction with our pipette tool and multiple decks. Duckweed roots are of interest to researchers [47], however root length measurements are more tedious to conduct compared to frond growth. Frond transfer techniques can be used in combination with both top and side cameras to develop an image processing pipeline for measuring root lengths. The base capabilities of the Duckbot might also be productively extended to other plants and small microorganisms. Integration of existing software into the Ducbot system is straightforward given our programmatic approach to machine control. Overall, we see the infrastructure presented here to be useful across several domains.

## Conclusion

We present the Duckbot, a system for automated imaging and manipulation of duckweed. We report on a case study of a niche laboratory workflow–duckweed growth assays–to explore the opportunities and limitations of laboratory automation using our system. We combine a set of interchangeable tools and associated control software to reproduce expected results. In the process, we contribute a set of design files and software alongside thorough documentation for use by other scientists. More broadly, we envision the tools discussed here to be a starting point for a growing community of scientists to develop and implement new tools for custom experiments. Developing a common ecosystem for scientists to leverage automation in domain-specific ways requires a heightened commitment to documentation and community building which we model in our resources for the Duckbot.
